# Synthesis and functionalization of NaGdF_4_:Yb,Er@NaGdF_4_ core–shell nanoparticles for possible application as multimodal contrast agents

**DOI:** 10.3762/bjnano.8.183

**Published:** 2017-09-01

**Authors:** Dovile Baziulyte-Paulaviciene, Vitalijus Karabanovas, Marius Stasys, Greta Jarockyte, Vilius Poderys, Simas Sakirzanovas, Ricardas Rotomskis

**Affiliations:** 1 Faculty of Chemistry and Geosciences, Vilnius University, Naugarduko str. 24, Vilnius LT-03225, Lithuania; 2 Biomedical Physics Laboratory, National Cancer Institute, Baublio str. 3b, Vilnius 2, Lithuania; 3 Department of Chemistry and Bioengineering, Vilnius Gediminas Technical University, Sauletekio Ave. 11, Vilnius, LT-10223, Lithuania; 4 Biophotonics group of Laser Research Center, Faculty of Physics, Vilnius University, Sauletekio Ave. 9, Vilnius LT-10222, Lithuania; 5 Institute of Chemistry, Center for Physical Sciences and Technology, Sauletekio Ave. 3, Vilnius, LT-10222, Lithuania

**Keywords:** cancer theranostics, core–shell structure, luminescence, multimodal, nanoparticles, upconverting nanoparticles, upconversion

## Abstract

Upconverting nanoparticles (UCNPs) are promising, new imaging probes capable of serving as multimodal contrast agents. In this study, monodisperse and ultrasmall core and core–shell UCNPs were synthesized via a thermal decomposition method. Furthermore, it was shown that the epitaxial growth of a NaGdF_4_ optical inert layer covering the NaGdF_4_:Yb,Er core effectively minimizes surface quenching due to the spatial isolation of the core from the surroundings. The mean diameter of the synthesized core and core–shell nanoparticles was ≈8 and ≈16 nm, respectively. Hydrophobic UCNPs were converted into hydrophilic ones using a nonionic surfactant Tween 80. The successful coating of the UCNPs by Tween 80 has been confirmed by Fourier transform infrared (FTIR) spectroscopy. Scanning electron microscopy (SEM), powder X-ray diffraction (XRD), photoluminescence (PL) spectra and magnetic resonance (MR) T1 relaxation measurements were used to characterize the size, crystal structure, optical and magnetic properties of the core and core–shell nanoparticles. Moreover, Tween 80-coated core–shell nanoparticles presented enhanced optical and MR signal intensity, good colloidal stability, low cytotoxicity and nonspecific internalization into two different breast cancer cell lines, which indicates that these nanoparticles could be applied as an efficient, dual-modal contrast probe for in vivo bioimaging.

## Introduction

Lanthanide-doped multimodal upconverting nanoparticles (UCNPs), which can convert near-infrared (NIR) radiation into visible light, have been extensively investigated due to the advantages associated with their unique optical properties [1]. Compared with traditional semiconductor quantum dots (QDs) or organic fluorophores, UCNPs show superior features such as sharp emission peaks, low toxicity, high photochemical stability, high resistance to photobleaching, and long emission lifetime [2–3]. As a unique class of luminescent phosphors, UCNPs show great promise in a broad range of applications ranging from bioimaging, biosensors, drug delivery, to photodynamic therapy [4–8]. Through combination with biologically active molecules, UCNPs could be multifunctional in both therapy and diagnostics (theranostics) [9]. However, biomedical applications require ultrasmall multifunctional nanoparticles to be hydrophilic, biocompatible and have intense upconversion emission and efficient paramagnetic properties. Hexagonal phase sodium gadolinium fluoride β-NaGdF_4_ is an ideal matrix for the creation optical/magnetic dual-modal bioprobes, but upconversion luminescence (UCL) efficiency of this host material is still low and needs to be improved. A major method to enhance the UCL intensity is to use a core–shell structure, where the nonactive shell protects the luminescent rare earth ions in the core from quenching caused by surface defects and organic ligands [10]. A wide variety of studies were performed to synthesize dual functional core–shell UCNPs [11–13]. However, it remains difficult to obtain hexagonal phase NaGdF_4_ (a host material exhibiting about an order of magnitude higher upconversion luminescence efficiency compared to cubic ones) with great optical and magnetic properties while maintaining a small size (<20 nm).

The next problem is that those nanoparticles are often synthesized in an organic phase and stabilized with hydrophobic ligands, such as oleic acid. Consequently, they can only be dispersed in nonpolar solvents (e.g., toluene, cyclohexane). In the past few years, several methods including surface silanization [14], ligand exchange [15], ligand oxidation [16], ligand removal [17], and amphiphilic polymer coating [18] have been developed in order to transfer nanoparticles with hydrophobic surfaces into aqueous media. Furthermore, the multimodal UCNP surface modification field still lacks reference materials and established protocols for functionalization and targeting. Some studies showed that the nonionic surfactant Tween 80 helps different nanoparticles (gold, silver and iron oxide) to become well-dispersed in aqueous solution even in the presence of biological molecules, such as different serum proteins [19–21]. However, information about Tween 80-coated gadolinium-based UCNPs behavior in biological systems and biocompatibility/nanotoxicity is still limited. The study of Cascales et al. showed that ultrasmall Yb:Er:NaGd(WO_4_)_2_ UCNPs could be successfully covered with Tween 80 and are internalized by human mesenchymal stem cells without triggering their metabolic activity, but still no information has been presented about uptake of these nanoparticles into different types of cancer cells [22]. Although different gadolinium chelates are widely used in clinics as contrast agents for magnetic resonance imaging (MRI), the literature for the last two years shows increased awareness of the effects of gadolinium toxicity [23–24]. Moreover, the possible influence of gadolinium-based UCNPs on cells is not yet investigated and understood.

In this work, we focus on studies of multimodal core–shell NaGdF_4_:Yb,Er coated with NaGdF_4_ (NaGdF_4_:Yb,Er@NaGdF_4_) UCNPs synthesis and demonstrate the effective surface modification method that uses a surfactant polysorbate 80 (Tween 80, polyoxyethylene sorbitan laurate). Hexagonal phase β-NaGdF_4_ was chosen as host lattice for its ability to combine optical and MRI. Tween 80 was used to make the UCNPs colloidally stable and dispersible in water while protecting the surface from nonspecific adsorption of biomolecules. Our results show that Tween 80-coated NaGdF_4_:Yb,Er@NaGdF_4_ core–shell nanoparticles exhibit excellent dispersibility in a biological medium and are photostable. We also do not observe any changes in the overall upconversion (UC) emission intensity of Tween 80-coated nanoparticles in comparison with oleic acid coated UCNPs. In addition, the nonspecific uptake and distribution of non-targeted Tween 80-coated UCNPs in human MCF-7 and MDB-MA-231 breast cancer cells was visualized by using confocal fluorescence microscopy. Our results showed that Tween 80-coated UCNPs exhibited low cytotoxicity even at a high-dose concentration.

## Results and Discussion

**Figure 1:**
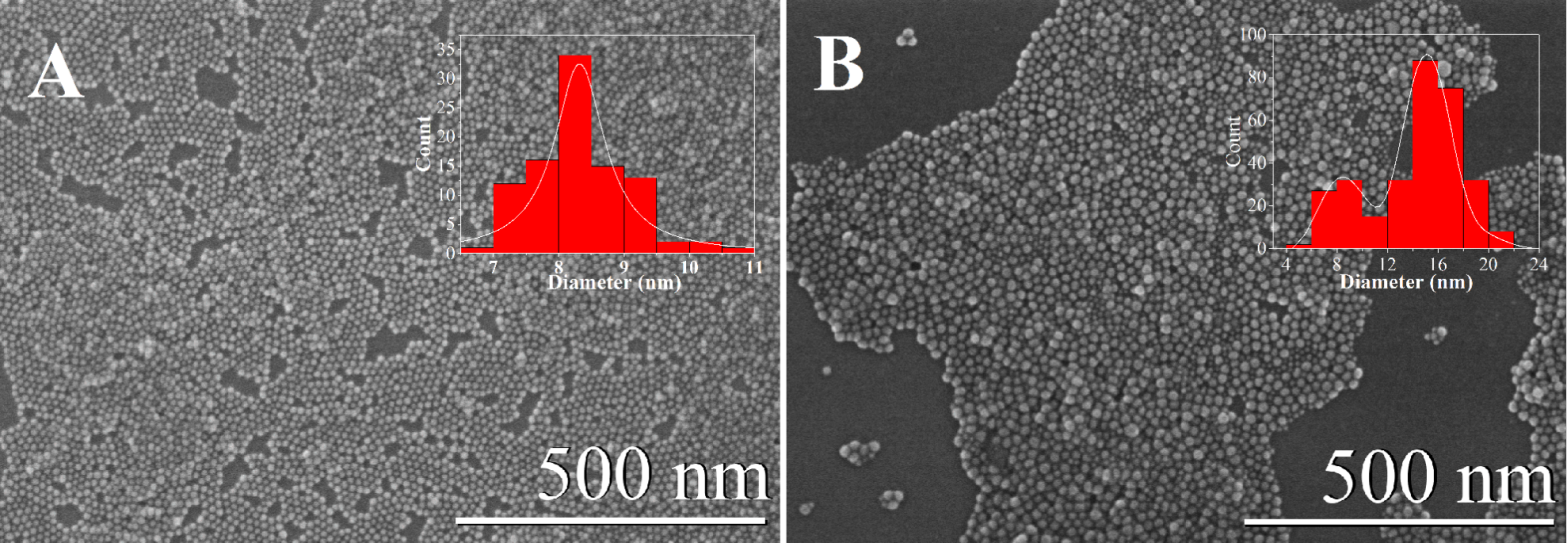
SEM images of the core NaGdF_4_:Yb,Er (A) and core@shell NaGdF_4_:Yb,Er@NaGdF_4_ (B) nanoparticles. The insets display the UCNP diameter distributions.

**Figure 2:**
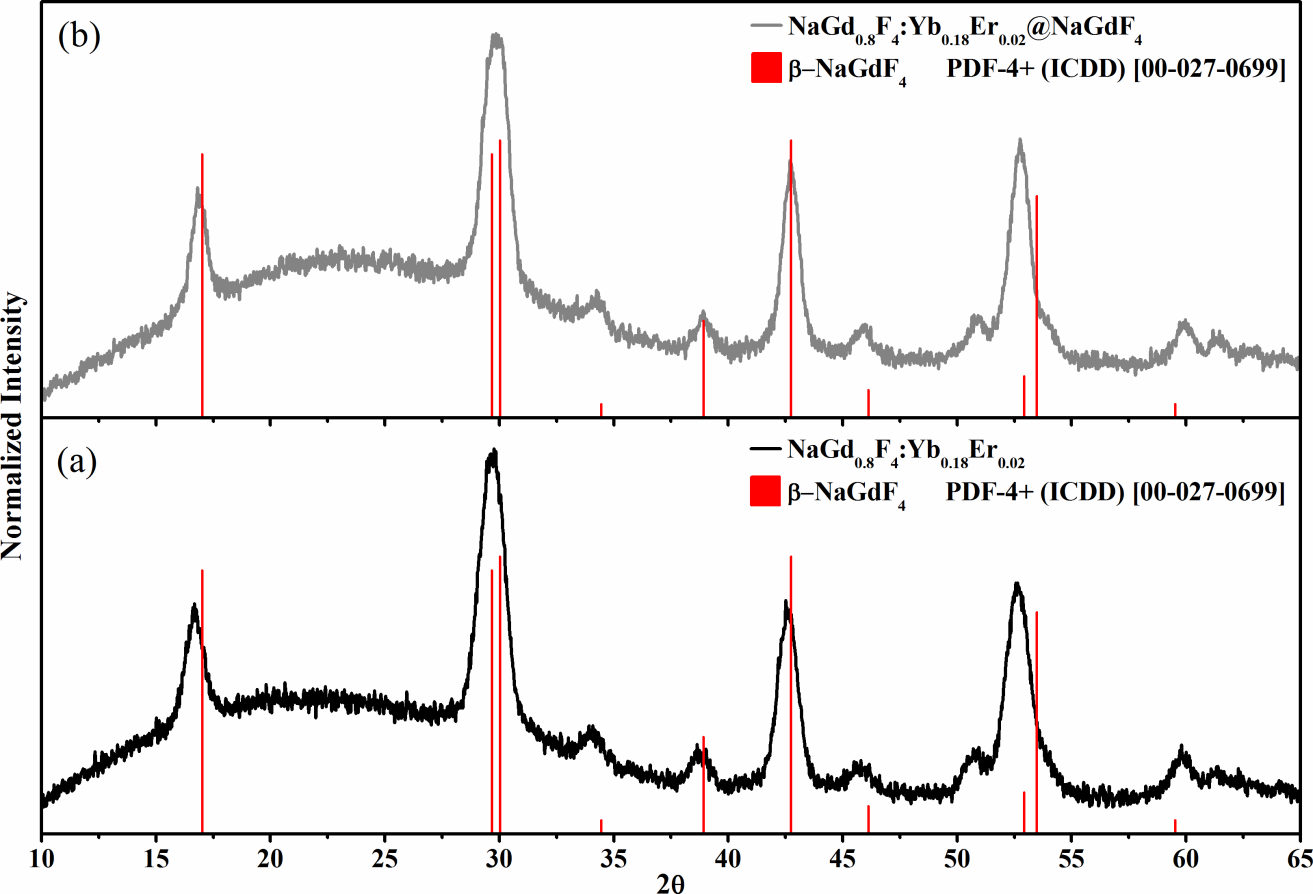
XRD pattern of NaGdF_4_:Yb,Er core only (a), and NaGdF_4_:Yb,Er@NaGdF_4_ core–shell (b) nanoparticles.

The SEM images of the NaGdF_4_:Yb,Er core and NaGdF_4_:Yb,Er@NaGdF_4_ core–shell nanoparticles are shown in Figure 1. Core nanoparticles are monodisperse, and have a spherical shape with an average diameter of approximately 8 nm with polydispersity index (PDI) of 1.02. The resulting core–shell nanoparticles are polydisperse and have an average diameter of ≈16 nm with PDI of 1.16. This indicates that polydispersity occurred from secondary nucleation during the shell growth process. However, an increase of the size suggests that the NaGdF_4_ has been successfully epitaxial grown on the NaGdF_4_:Yb,Er core nanoparticles. The diffraction peaks of the core (Figure 2a) and core–shell (Figure 2b) nanoparticles can be indexed as pure hexagonal β-NaGdF_4_ phase (JCPDS, Card No. 27-0699), indicating no change in the crystalline phase during the shell growth.

The as-obtained core and core–shell UCNPs were hydrophobic as they were stabilized by oleic acid molecules. In this work, hydrophobic core and core–shell nanoparticles were converted into hydrophilic ones using a nonionic surfactant Tween 80.

**Figure 3:**
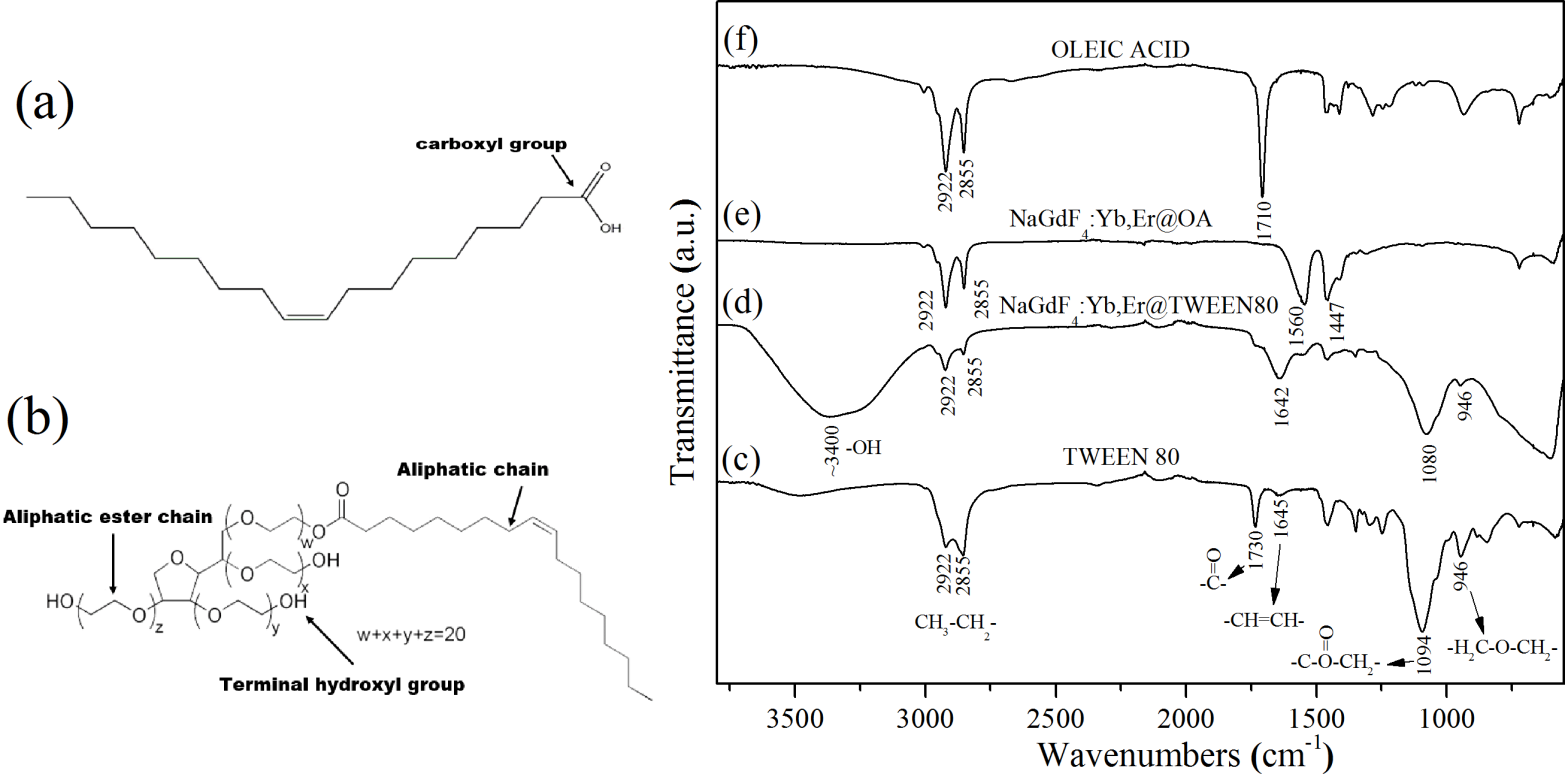
The structure of (a) oleic acid (OA) and (b) Tween 80. FTIR spectra of (c) pure Tween 80, (d) NaGdF_4_:Yb,Er@Tween80, (e) NaGdF_4_:Yb,Er@OA, and (f) pure OA.

The presence of the Tween 80 coating was verified by comparing its FTIR spectra to that of pure oleic acid, oleate ligands coated particles, pure Tween 80, and the final coated nanoparticles (Figure 3). NaGdF_4_:Yb,Er UCNPs prepared in the presence of oleic acid shows characteristic absorption peaks of oleate ligands. The absorption peak at 1710 cm^−1^ (Figure 3f) corresponds to the stretching vibration of C=O in pure oleic acid (Figure 3a) which is replaced by two carboxylate stretching bands (1560 and 1447 cm^−1^ in Figure 3e), which indicates oleate ligand adsorption on the UCNP surface.

Tween 80 is composed of three building blocks: aliphatic ester chains, three-terminal hydroxyl groups and an aliphatic chain (Figure 3b). The aliphatic chain can be adsorbed on the hydrophobic surface by hydrophobic interactions of UCNPs as synthesized in oleic acid [25]. The strong band around 3400 cm^−1^ can be assigned to the O–H stretching vibrations (Figure 3d) from terminal hydroxyl groups of Tween 80 (Figure 3b) and the remaining moisture in the samples. The bands centred at 2922 and 2855 cm^−1^ are associated with the asymmetric (νas) and symmetric (νs) stretching vibrations of methylene (–CH2), respectively. The adsorption peaks at 1730 and 1094 cm^−1^ are attributed to the ester group stretching. The band at 946 cm^−1^ is present, which corresponds to the ether bond from the aliphatic ester chains (Figure 3c). The FTIR data of UCNPs@Tween80 (Figure 3d) is highly comparable with that of pure Tween 80 (Figure 3c), indicating that the Tween 80 was successfully coated onto the UCNPs. Additionally, dynamic light scattering (DLS) was employed to measure the hydrodynamic diameter of Tween-coated UCNPs in the cell culture medium as well as their surface zeta potential. The measured mean hydrodynamic diameter of the Tween-coated core NaGdF_4_:Yb,Er UCNPs was 38 nm and the core–shell NaGdF_4_:Yb,Er@NaGdF_4_ particles was 48 nm. The zeta potential of Tween 80-coated core nanoparticles was about 26 mV and for core–shell nanoparticles it was slightly higher at about 33 mV. More detailed information about the DLS results is presented in the Supporting Information File 1.

**Figure 4:**
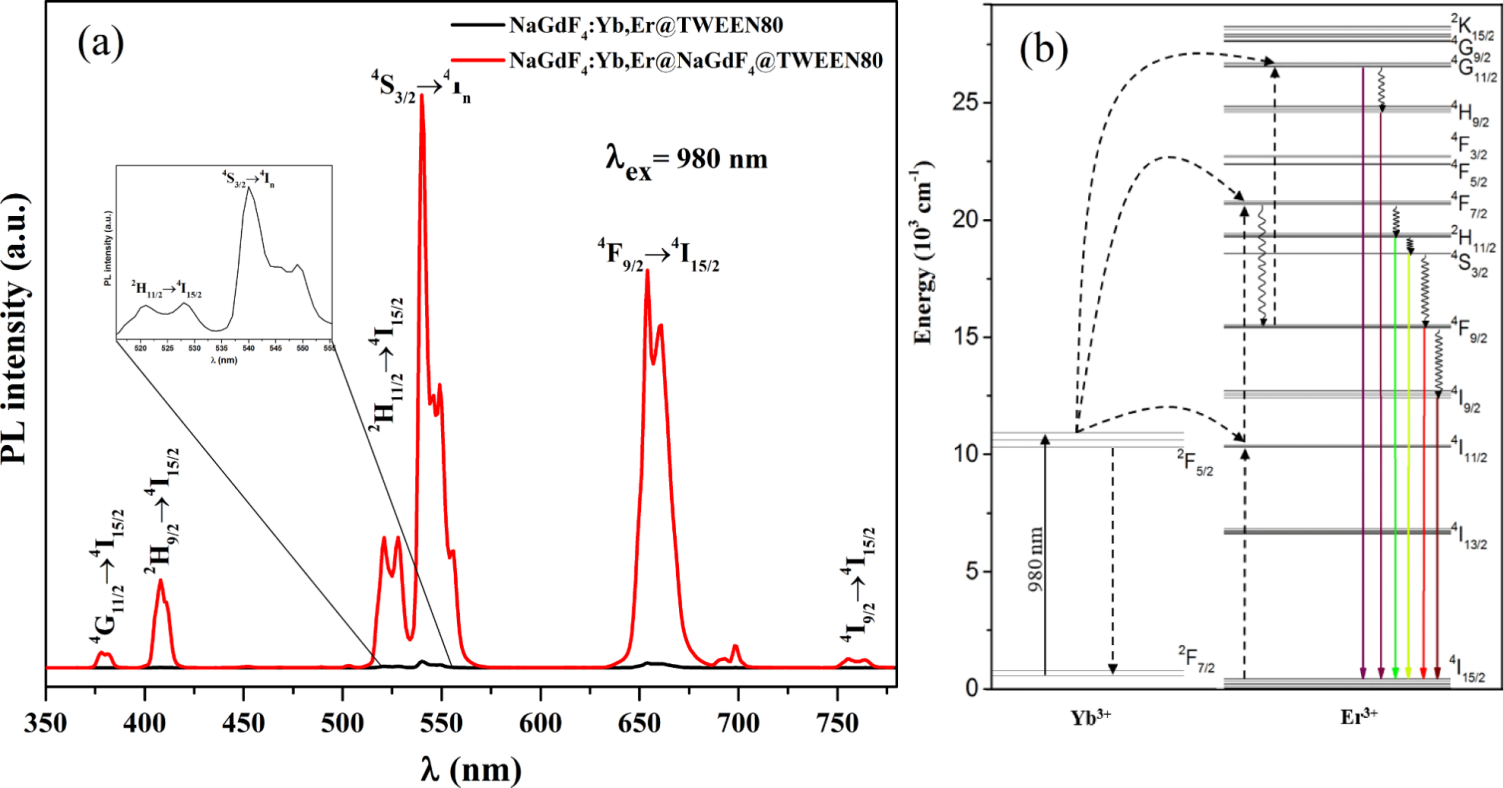
(a) Upconversion luminescence spectra of Tween 80-coated UCNPs upon 980 nm excitation [28] and (b) energy level diagram of Yb^3+^ and Er^3+^ ions.

The upconversion emission spectra of different NaGdF4:Yb,Er@NaGdF4@Tween80 core–shell and NaGdF_4_:Yb,Er@Tween80 core nanoparticles dispersed in water are shown in Figure 4a. The major emissions located at 381, 408, 521, 540, 654 and 756 nm can be attributed to radiative transitions from ^4^G_11/2_^2^H_9/2_, ^2^H_11/2_, ^4^S_3/2_, ^4^F_9/2_ and ^4^I_9/2_ levels to the ^4^I_15/2_ level of Er^3+^ (Figure 4b), respectively. The comparison with the core-only nanoparticles showed that coating the NaGdF_4_:Yb^3+^,Er^3+^ core with a shell that has the same crystal lattice structure reduce the effects of luminescence quenching from the addition of ligands and/or surface defects and therefore a significant increase in the UCL can be observed. For the core-only nanoparticles, lanthanide dopants are exposed to surface deactivations owing to the high surface-to-volume ratio at the nanometer dimension, thus yielding UCL at low efficiency. The integrated intensity (521 nm) of the core–shell NaGdF_4_:Yb,Er@NaGdF_4_ nanoparticles was estimated to be about two magnitudes higher than the core-only NaGdF_4_:Yb,Er UCNPs. The results indicate that the core–shell structure can effectively spatially isolate lanthanide dopants from being quenched, and also negate the influence of surface defects. The results correlate well with what is presented in the literature. Yi et al. reported that the UC emissions of hexagonal phase NaYF_4_:Yb^3+^,Er^3+^ were enhanced by as much as seven times by growth of a 2 nm layer of NaYF_4_ [26]. In a later publication, the same conclusion was independently verified in core–shell UCNPs of NaGdF_4_:Yb^3+^,Tm^3+^@NaGdF_4_ and KGdF_4_:Yb^3+^,Tm^3+^@KGdF_4_ when compared to the core under 980 nm excitation [11–12,27].

**Figure 5:**
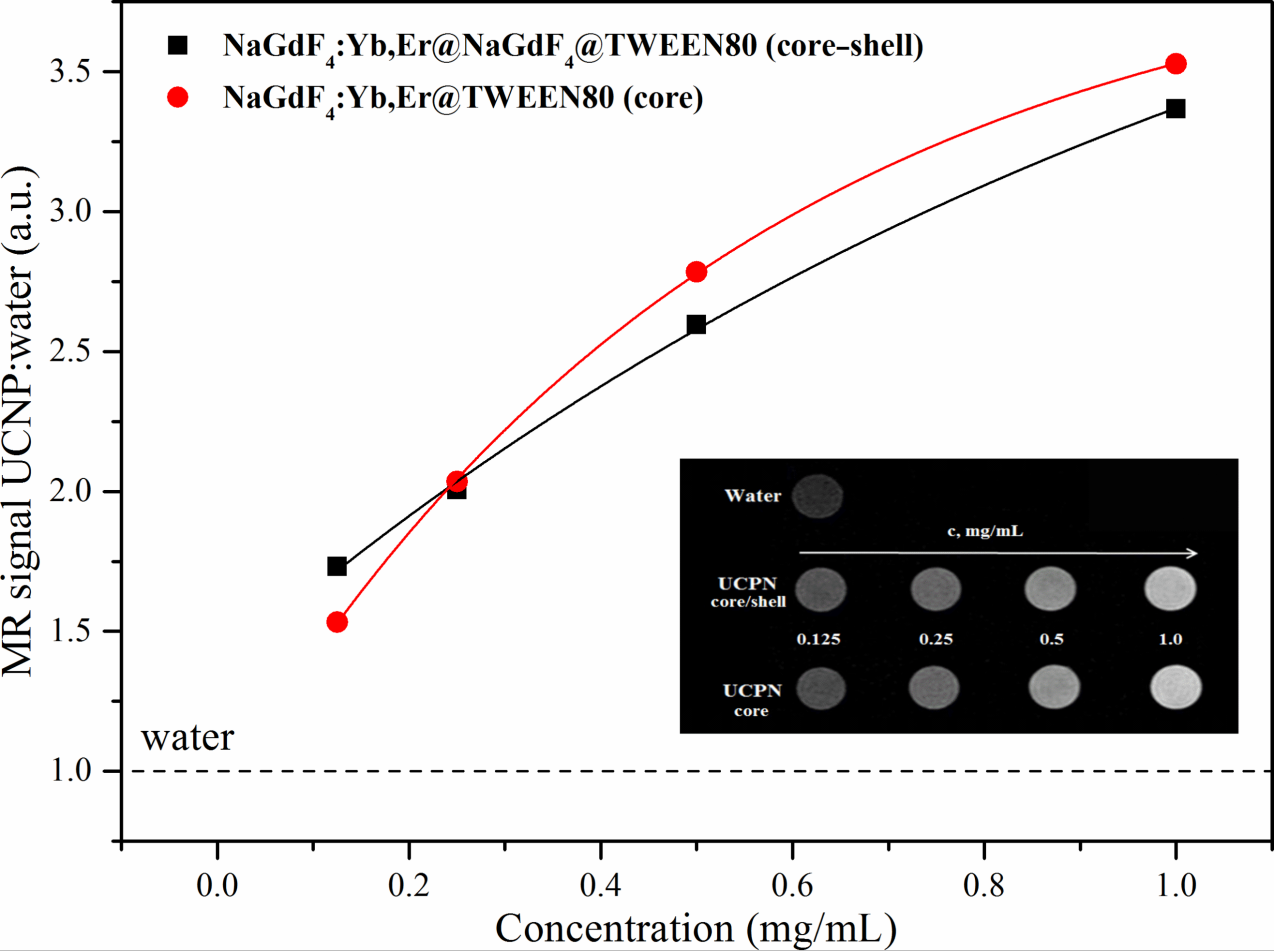
Magnetic resonance (MR) signal intensity (SI) plot of core (red dots) and core–shell (black squares) UCNPs of different concentrations of aqueous solutions. Water SI is marked as a dashed line as a reference; Inset: T1-weighted MR in vitro images of core and core–shell UCNPs at different concentrations of aqueous solutions.

As shown in Figure 5 (inset), a positive enhancement for the magnetic resonance (MR) signal was observed for all the UCNPs samples when compared to water. Moreover, with the increase of the concentration of UCNPs, the T1-weighted MRI signal intensity (SI) continuously increased, resulting in brighter images for both types of UCNPs. The MR SI values of UCNPs are presented in Figure 5. The maximum MR signal enhancement was of approximately 3.5-fold compared with the reference. There was no significant difference observed in MR signal enhancement between the core and core–shell UCNPs. Therefore, it can be concluded that the UCNP coating does not affect the favorable MRI properties of UCNPs. That signifies that the Gd^3+^ ions in the shell of the UCNPs are the major contributors toward the relaxation of water protons, and the UCNP core does not show any significant effect towards relaxivity enhancement. However, it has been shown in the literature that reduced water access to the Gd^3+^ ions may yield reduced values for MR signal enhancement [29–30]. These observations indicate that both core and core–shell UCNPs could be applied as efficient MRI contrast agents as they both present enhanced MR signal intensity.

**Figure 6:**
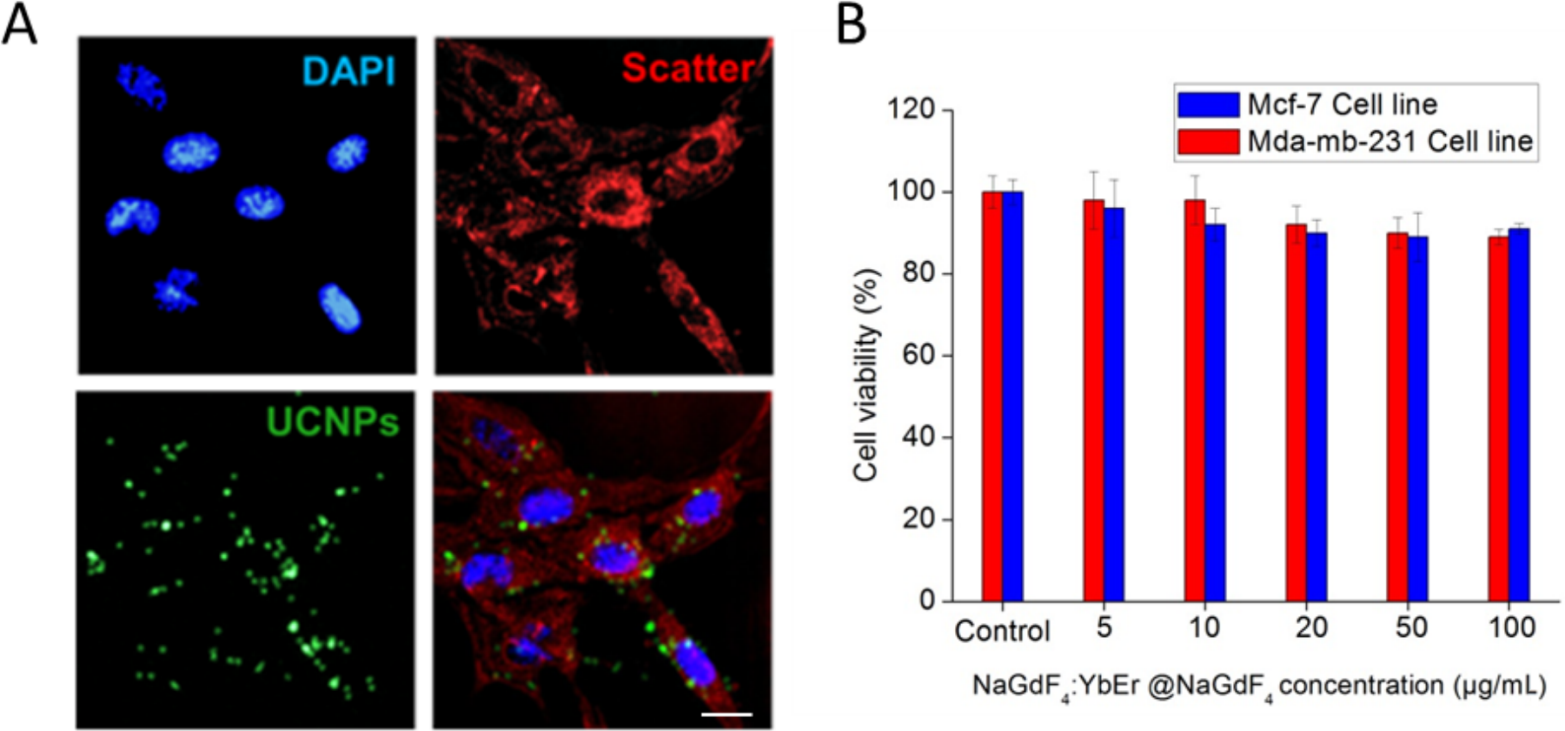
A) Confocal images of MDA-MB-231 cells after 24 h treatment with Tween 80-coated core–shell UCNPs (10 µg/mL); UCNPs are green, DAPI staining is blue, the red color represents excitation scattering from intracellular structures. Scale bar equals 10 µm. B) Viability of MCF-7 and MDA-MB-213 cells, treated with different concentrations of UCNPs for 24 h. Toxicity of UCNPs was investigated using XTT cell viability assay.

The as-prepared Tween 80-coated core–shell NaGdF_4_:Yb,Er@NaGdF_4_ nanoparticles were studied to evaluate their application to biological imaging using MDA-MB-231 breast cancer cells. The confocal image of MDA-MB-231 breast cancer cells after 24 h incubation with UCNPs is shown in Figure 6A. The scatter of excitation light by intracellular cell structures was marked with red color. This was obtained by excitation at 514 nm and detected at 500–530 nm. Tween 80-coated core–shell UCNPs were marked with green color (excitation was continuous wave at 980 nm and detection at 500–530 nm). The cell nuclei were labeled with DAPI and imaged using an excitation of 405 nm and detected at using a bandpass filter with a center wavelength of 450 nm and bandwidth of 35 nm. As seen from Figure 6A, the luminescence of the UCNPs came from the intracellular region, suggesting that Tween 80-coated nanoparticles were non-specifically internalized into cells and concentrated within the cytoplasm. The similar localization of Tween 80-coated nanoparticles was observed in MCF-7 cells as well. The same results of endocytic NP accumulation in cells was demonstrated in different studies with UCNPs [31], quantum dots [32], magnetic nanomaterials [33] and noble metal nanoparticles [34].

Cell viability assay XTT was performed to measure the cellular metabolic activity of human breast cancer MCF-7 and MDA-MB-231 cell lines after 24 h treatment with core–shell Tween 80-coated UCNPs (Figure 6B). Untreated cells were used as a control group. After 24 h of incubation in the UCNP concentration range from 5 to 100 μg/mL, the viability of human breast cancer MCF-7 cells remained over 92–100% and the viability of MDA-MB-231 cells remained 85–93%. These results clearly express that core–shell gadolinium-based UCNPs have low cytotoxicity and are in good agreement with previous studies [35–36].

## Conclusion

In summary we have successfully synthesized ultrasmall, monodisperse, hexagonal phase core NaGdF_4_:Yb,Er nanoparticles and polydisperse, core–shell NaGdF_4_:Yb,Er@NaGdF_4_ nanoparticles.

Oleate-capped core NaGdF_4_:Yb,Er nanoparticles and core–shell NaGdF_4_:Yb,Er@NaGdF_4_ nanoparticles were successfully transferred to aqueous solutions after surface modification with the surfactant Tween 80. The core–shell UCNPs presented enhanced upconversion intensity and MR signal intensity, which indicates that these nanoparticles could be applied as an efficient dual optical, MRI contrast agent. Moreover, an in vitro uptake and cytotoxicity evaluation study showed that the UCNPs internalized into breast cancer cell lines and possessed low cytotoxicity and good biocompatibility. All these findings indicate that Tween 80-coated NaGdF_4_:Yb,Er@NaGdF_4_ UCNPs are a promising nanomaterial platform for imaging and detection in oncology.

## Experimental

**Materials:** All of the chemicals used in our experiments were of analytical grade and used without further purification. Ln oxides (Ln_2_O_3_, 99.99%, Ln: Gd, Yb, Er) were obtained from Treibacher Industrie AG (Germany). Oleic acid (OA, 90%) was purchased from Fisher Scientific, 1-octadecene (ODE, 90%) was obtained from Sigma-Aldrich. Tween 80 (polysorbate 80) was purchased from Merck Millipore. Other chemicals including hydrochloric acid, sodium hydroxide, ammonium fluoride, methanol, chloroform, cyclohexane and acetone were obtained from Reachem Slovakia.

**Synthesis of core β-NaGdF_4_:Yb,Er nanoparticles:** The synthesis of β-NaGdF_4_:Yb,Er NPs was developed via a modified procedure from the literature [11]. In a typical experiment, 1.6 mmol Gd_2_O_3_, 0.36 mmol Yb_2_O_3_ and 0.04 mmol Er_2_O_3_ were dissolved in HCl at an elevated temperature (≈80 °C) to prepare the rare earth chloride stock solution. Metal chlorides were mixed with 12 mL oleic acid (OA) and 30 mL 1-octadecene (ODE) in three-neck round-bottom flask and then heated to 150 °C for 40 min. 10 mL of methanol solution containing NaOH (5 mmol) and NH_4_F (8 mmol) was slowly introduced and the solution was stirred at 50 °C for 30 min. After the methanol was evaporated, the solution was heated to 300 °C for 1 h under argon atmosphere. The resultant nanoparticles were precipitated by hexane/acetone (1:4 v/v), collected by centrifugation, washed with acetone and DI water several times, and finally redispersed in cyclohexane.

**Synthesis of core–shell β-NaGdF_4_:Yb,Er@NaGdF_4_ nanoparticles:** The subsequent deposition of the NaGdF_4_ shell followed a similar process for the preparation of NaGdF_4_:Yb,Er core particles. 1 mmol Gd_2_O_3_ was dissolved in HCl at an elevated temperature (≈80 °C) to prepare a 2 mmol gadolinium chloride stock solution. 2 mmol gadolinium chloride was added to a three-neck round-bottom flask containing 8 mL OA and 30 mL ODE and then heated to 150 °C for 40 min under argon atmosphere to form a homogeneous solution and then cooled to room temperature. 10 mL of cyclohexane solution of 0.66 mmol NaGdF_4_:Yb,Er nanoparticles was added dropwise into the solution. The mixture was degassed at 100 °C for 10 min to remove cyclohexane and cooled to room temperature. Then 10 mL methanol solution of NaOH (5 mmol) and NH_4_F (8 mmol) was added and stirred at 50 °C for 30 min. After the methanol evaporated, the solution was heated to 300 °C for 1 h under argon atmosphere. The resultant core–shell nanoparticles were precipitated by hexane/acetone (1:4 v/v), collected by centrifugation, washed with acetone and DI water several times, and finally redispersed in cyclohexene.

**Figure 7:**
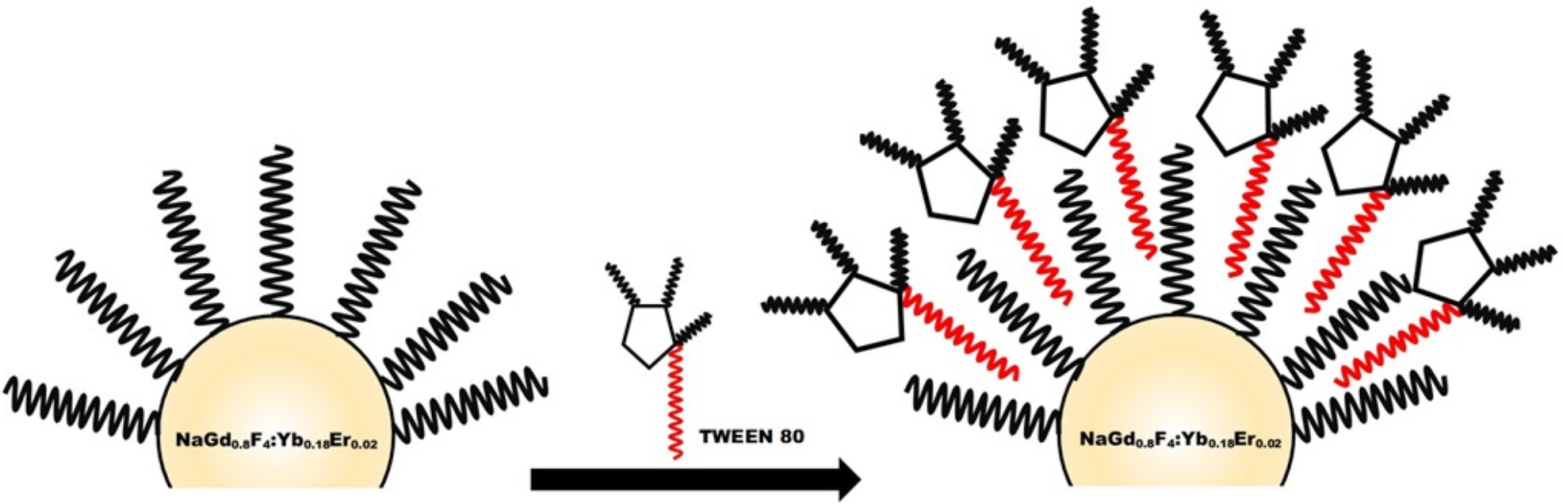
Formation of water-soluble core and core–shell UCNPs by coating with Tween 80.

**Tween modification of oleate-capped β-NaGdF_4_:Yb,Er and β-NaGdF_4_:Yb,Er@NaGdF_4_ nanoparticles:** The surface modification of β-NaGdF_4_:Yb,Er and β-NaGdF_4_:Yb,Er@NaGdF_4_ nanoparticles was carried out following a literature protocol with slight modifications [37]. In a typical experiment, 400 μL of Tween 80 was added into a round-bottom flask containing ≈20 mg of β-NaGdF_4_:Yb,Er (β-NaGdF_4_:Yb,Er@NaGdF_4_) and 8 mL of CHCl_3_, and the solution was stirred for 1 h at room temperature. 20 mL of deionized water was poured in the flask and the dispersion was kept in a 80 °C water bath for 3 h. During this period, the CHCl_3_ was evaporated and the hydrophobic UCNPs were gradually converted into hydrophilic ones. A principle mechanism by which the Tween 80 surfactant stabilizes the UCNPs is shown in Figure 7.

**Characterization:** The polydispersity index of UCNPs was calculated by finding the weight (*D*_w_) and number-average diameter (*D*_n_) ratio using the following equations:

[1]
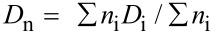


[2]
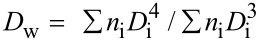


where *n*_i_ and *D*_i_ are the number and diameter of the particle, respectively.

Dynamic light scattering (DLS) was used to determine the hydrodynamic particle diameter and zeta potential. These experiments were performed with Brookhaven ZetaPALS zeta potential analyzer (Brookhaven Instruments, USA). Powder X-ray diffraction (XRD) analysis has been carried out by employing a Rigaku MiniFlex II diffractometer working in the Bragg–Brentano (θ/2θ) geometry. The data were collected within a 2θ angle from 10° to 65° at a step of 0.01° and scanning speed of 10 °/min using the Ni-filtered Cu Kα line. The particle morphology was characterized using a field emission scanning electron microscope (SU-70 Hitachi, FE-SEM) at an acceleration voltage of 10 kV. The UC luminescence spectra were recorded using an Edinburgh Instruments FLS980 spectrometer equipped with a double emission monochromator, a cooled (−20 °C) single-photon counting photomultiplier (Hamamatsu R928), and a 1 W continuous wavelength 980 nm laser diode. The emission slit was set to 1 nm, the step size was 1 nm, and the integration time was 0.1 s with 5 scans to gain more intensity. The emission spectra were corrected by a correction file obtained from a tungsten incandescent lamp certified by National Physics Laboratory, UK. The measurements were performed in standard 1 cm quartz cuvettes at room temperature. Fourier transform infrared (FTIR) spectra were recorded on an infrared spectrometer (Perkin Elmer Spectrum).

**Cell culturing and imaging:** Human breast cancer cell lines MDA-MB-231 and MCF-7 were obtained from the American Type Culture Collection (ATCC HTB-26™; ATCC HTB-22™). MDA-MB-231 and MCF-7 cells were cultured in cell growth medium (DMEM, Gibco, US), supplemented with 10% (v/v) fetal bovine serum (FBS) (Gibco, US), 100 U/mL penicillin and 100 mg/mL streptomycin. The cells were maintained at 37 °C in a humidified atmosphere containing 5% of CO_2_. The cells were routinely subcultured 2–3 times a week in 25 cm^2^ culture dishes. Prior to the UCNP experimentation, the uptake cells were seeded and allowed to grow for 24 h and then treated with 10 µg/mL of Tween 80-coated core–shell UCNPs for 24 h. Then the cells were fixed with 4% paraformaldehyde and stained with DAPI. The high-resolution imaging system for UCNP imaging was based on a confocal microscopy system Nikon C1si (Japan). A 980 nm continuous wave laser with an intensity control module was introduced into the confocal microscopy system for excitation of samples in the NIR spectral region. 450/35 nm, 515/30 nm and 605/75 nm band pass filters (where the first value is the center/peak wavelength and the second refers to the bandwidth of the filter) were used to block detectors from reflected and scattered NIR light.

**Cell viability assay:** MCF-7 and MDA-MB-231 human breast cancer cells were seeded on a 96-well plate at a density of 20,000 cells/well. After 24 h, the old medium was replaced with a fresh medium containing 5, 10, 20, 50 and 100 µg/mL core–shell UCNPs. 12 wells were left without upconverting particles to serve as the control group. After 24 h of treatment, the cell growth medium with nanoparticles was aspirated and cells were washed with DPBS (pH 7.0) three times. To prepare an XTT solution, 0.1 mL activation solution (*N*-methyl dibenzopyrazine methyl sulfate) was mixed with 5 mL XTT reagent (tetrazolium derivative). 100 µL of a fresh medium and 50 μL of the reaction solution were added to each well and the plate was incubated for 5 h in an incubator at 37 °C. After incubation, optical density values at 490 nm were measured using the Biotek (USA) microplate reader. Values obtained from measuring optical density were recalculated as percentage values of viability. The absorbance value of the control group was set to 100% and the rest of the values were recalculated accordingly.

**in vitro MR imaging:** The MR signal enhancement measurements were carried out on a 1.5 T clinical MRI scanner (Achieva, Philips Medical Systems, Best, The Netherlands) in conjunction with a Sense Flex-M coil (Philips Medical Systems, Best, The Netherlands). Dilutions of core and core–shell UCNPs (0.125, 0.25, 0.5, 1.0 mg/mL) in deionized water were prepared for T1-weighted MR imaging and T1-weighted contrast enhancement. A series of aqueous solutions of UCNPs were placed in an array of 2.0 mL Eppendorf tubes with the order of UCNP concentrations and deionized water (0 mg/mL) was used as the reference. The parameters for T1-weighted MR imaging sequence was set as follows: echo time (TE) = 15.0 ms, repetition time (TR) = 500 ms, number of averages (NSA) = 8, matrix = 1024 × 1024, FOV = 200 × 200 mm, and slice thickness = 1.5 mm. The MR signal intensity (SI) in the tubes was determined by the average intensity in the defined regions of interests (ROIs). The resulting SI values in ROIs were plotted as a ratio of UCNP:water against the concentration of UCNPs.

**Statistical analysis:** Data are shown as the representative result or as mean of at least three independent experiments ±SD. Statistical analyses were performed using the two-tailed Student’s *t*-test; differences were considered significant at *p* < 0.05.

## Supporting Information

File 1 The hydrodynamic particle size and zeta potential.
The results representing hydrodynamic size distribution of UCNPs and their zeta potential that were measured using dynamic light scattering method (DLS).
