# Supplementary material for: Synthesis and functionalization of NaGdF4:Yb,Er@NaGdF4 core–shell nanoparticles for possible application as multimodal contrast agents
Source: Beilstein J Nanotechnol. 2017 Sep 1;8:1815–24. doi: 10.3762/bjnano.8.183 (PMC5588608; doi:10.3762/bjnano.8.183)
Supplement: File 1 — The hydrodynamic particle size and zeta potential. The results representing hydrodynamic size distribution of UCNPs and their zeta potential that were measured using dynamic light scattering method (DLS). [file Beilstein_J_Nanotechnol-08-1815-s001.pdf]

Supporting Information

for

Synthesis and functionalization of $NaGdF_4$:Yb,Er@$NaGdF_4$ core–shell nanoparticles for possible application as multimodal contrast agents

Dovile Baziulyte-Paulaviciene[1], Vitalijus Karabanovas[2,3*,§], Marius Stasys[2,4], Greta Jarockyte[2,4], Vilius Poderys[2], Simas Sakirzanovas[1,5] and Ricardas Rotomskis[2,4]

Address: [1]Faculty of Chemistry and Geosciences, Vilnius University, Naugarduko str. 24, Vilnius LT-03225, Lithuania, [2]Biomedical Physics Laboratory, National Cancer Institute, Baublio str. 3b, Vilnius 2, Lithuania, [3]Department of Chemistry and Bioengineering, Vilnius Gediminas Technical University, Sauletekio Ave. 11, Vilnius, LT-10223, Lithuania, [4]Biophotonics group of Laser Research Center, Faculty of Physics, Vilnius University, Sauletekio Ave. 9, Vilnius LT-10222, Lithuania and [5]Institute of Chemistry, Center for Physical Sciences and Technology, Sauletekio Ave. 3, Vilnius, LT-10222, Lithuania

Email: Vitalijus Karabanovas - vitalijus.karabanovas@nvi.lt

* Corresponding author

[§] Tel. +370 5 2190902

The hydrodynamic particle size and zeta potential

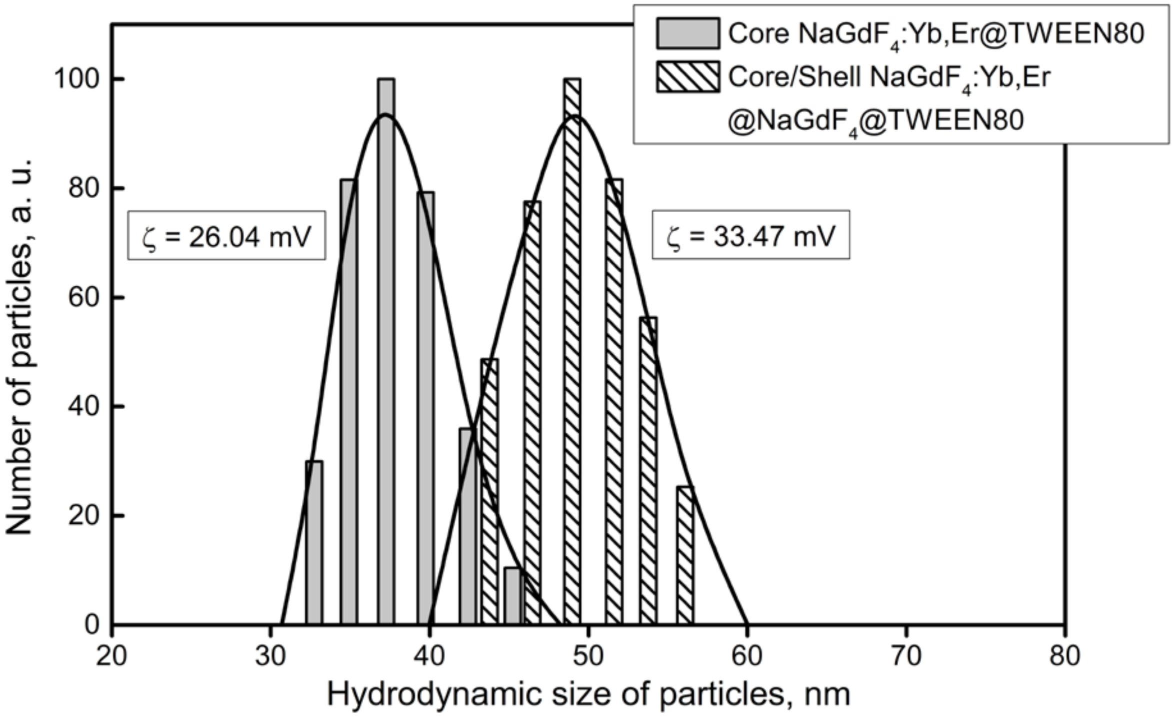

Figure S1: Hydrodynamic size distribution of core-only and core-shell UCNPs and their zeta potential in the cell culture medium.
